# Multi-Sensor Measurement of Physical Behavior in the Finnish Retirement and Aging Study

**DOI:** 10.1093/geroni/igaa057.2757

**Published:** 2020-12-16

**Authors:** Sari Stenholm

**Affiliations:** University of Turku, Turku, Finland

## Abstract

Retirement is a life transition that is accompanied by changes in time use and removal of work-related exposures. The Finnish Retirement and Aging study (FIREA) was established in 2013 to examine changes in 24-hour physical behavior, lifestyle factors, health and functioning by following aging workers annually from full-time work to retirement. FIREA activity substudy includes 1200 participants (mean age 63.2 years, 85% women) and their physical behavior has been measured annually with multiple accelerometers (wrist ActiGraph, thigh Axivity and hip SenseDoc including GPS). The mean number of measurement days is seven at each measurement wave with a valid wake wear time 16.0 hours before retirement and 15.6 hours after retirement. Currently, information is available from 3-5 measurement waves. Transition to retirement induced changes in 24-h physical behavior towards increased sleep and less physical activity among women, especially in those retiring from manual occupations.

